# An Eye to Business

**Published:** 1891-05

**Authors:** 


### An Eye to Business.

A certain doctor, who was noted for a keen eye to business, was driving along the street of a country town, when his horse took fright and ran away. He was thrown violently out of his trap and rendered senseless. Presently he recovered a little from his unconsciousness, and, noticing the crowd which had gathered about him, asked, "What's the matter, gentlemen? Anyhody hurt? I am Dr. B——. Can I be of any service?"

